# Bedaquiline Resistance after Effective Treatment of Multidrug-Resistant Tuberculosis, Namibia

**DOI:** 10.3201/eid3003.240134

**Published:** 2024-03

**Authors:** Gunar Günther, Lusia Mhuulu, Azaria Diergaardt, Viola Dreyer, Maria Moses, Kaarna Anyolo, Nunurai Ruswa, Mareli Claassens, Stefan Niemann, Emmanuel Nepolo

**Affiliations:** Inselspital Bern, Bern University Hospital, University of Bern, Bern, Switzerland (G. Günther);; University of Namibia, Windhoek, Namibia (G. Günther, L. Mhuulu, A. Diergaardt, M. Claassens, S. Niemann, E. Nepolo);; Katutura State Hospital, Windhoek, Namibia (G. Günther, M. Moses, K. Anyolo);; Research Center Borstel, Borstel, Germany (V. Dreyer, S. Niemann);; German Center for Infection Research, Partner Site Hamburg-Lübeck-Borstel-Riems, Borstel (V. Dreyer, S. Niemann);; National Tuberculosis and Leprosy Programme, Windhoek, Namibia (N. Ruswa)

**Keywords:** tuberculosis, TB, tuberculosis and other mycobacteria, bacteria, respiratory infections, bedaquiline, antimicrobial resistance, Namibia, MDR TB

## Abstract

Bedaquiline is currently a key drug for treating multidrug-resistant or rifampin-resistant tuberculosis. We report and discuss the unusual development of resistance to bedaquiline in a teenager in Namibia, despite an optimal background regimen and adherence. The report highlights the risk for bedaquiline resistance development and the need for rapid drug-resistance testing.

The development of bedaquiline, and its inclusion in first-line treatment of rifampin-resistant (RR) and multidrug-resistant (MDR, resistance to isoniazid and rifampin) tuberculosis (TB), along with linezolid, pretomanid, and moxifloxacin, the BPaL(M) regimen, has transformed the management of drug-resistant TB (*1*). The World Health Organization (WHO) began recommending BPaL(M) in 2022. However, recent studies have reported the emergence of bedaquiline resistance, which suggests that BPaL(M) may be unable to prevent bedaquiline resistance at population level (*2*,*3*). The mechanisms underlying the selection and spread of bedaquiline resistance are not yet well understood. We describe bedaquiline resistance evolution in a patient with MDR TB who had extensive bilateral pulmonary infiltrates despite a regimen of 6 effective drugs.

## The Study

**Figure 1:**
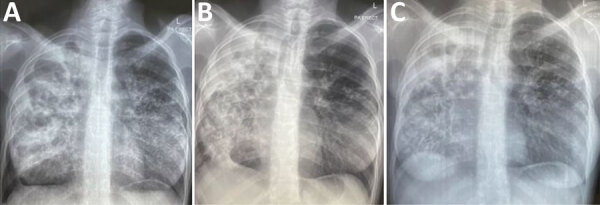
Chest radiographs showing lungs of patient in Namibia who developed bedaquiline resistance. A) At treatment initiation; B) at culture conversion; C) 3 months after conversion.

**Figure 2:**
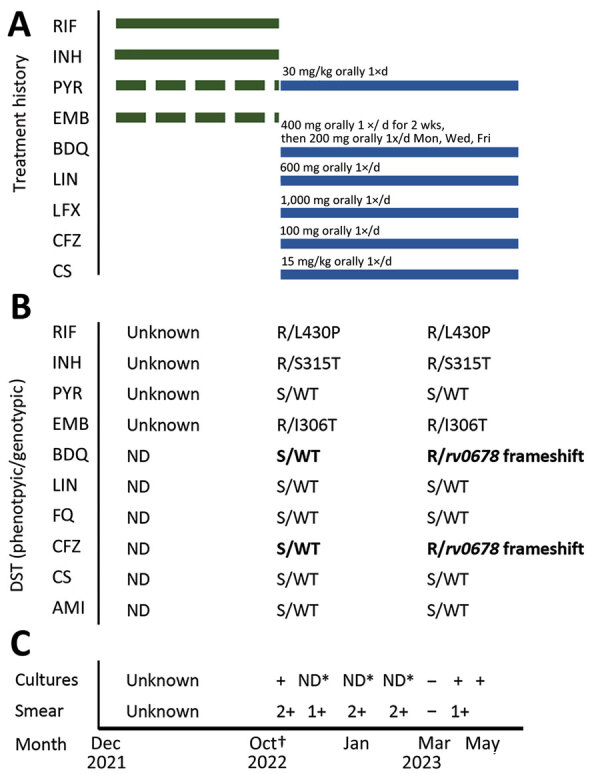
Timeline for a case of tuberculosis in a patient in Namibia whose infection became drug resistant after effective treatment. The case was originally diagnosed and treated beginning in December 2021. Interruptions in treatment were caused by stockout. Second-line drugs were not used. Full detailed treatment history is unknown. The patient sought care in Namibia in September 2022; we diagnosed MDR TB in October 2022. Treatment failed and *rv0678* mutation was identified in a culture from June 2023. A) Patient’s treatment history. Green bars represent treatment of drug-susceptible TB; blue bars represent treatment of MDR TB. B) Evolution of phenotypic and genotypic drug susceptibility testing with resistance-associated variants using Deeplex Myc TB (https://www.deeplex.com). Testing was done at time of diagnosis of MDR TB and after culture reversion. Bold text de novo indicates mutations. Months show time of specimen collection. C) Culture and smear test results. Asterisks indicate that tests were not done because of stockouts. Months show time of specimen collection. Dagger indicates start of MDR TB treatment. AMI, amikacin; BDQ, bedaquiline; CFZ, clofazimine; CS, cycloserine; DS, drug-susceptible; EMB, ethambutol; FQ, fluoroquinolones; INH, isoniazid; LIN, linezolid; LFX, levofloxacin; MDR, multidrug-resistant; ND, not done; PYR, pyrazinamide; R, resistant; RIF, rifampin; S, susceptible; TB, tuberculosis; WT, wild type.

In October 2022, a 16-year-old HIV-negative female patient sought care at a clinic in Windhoek, Namibia. She was severely underweight (BMI 16 kg/m^2^) and had radiologic findings of extensive, bilateral destruction of the lung parenchyma (Figure 1). The patient reported treatment for drug-sensitive TB with a standard first-line regimen since December 2021 in neighboring Angola, but she had treatment interruptions caused by stockout (Figure 2, panel A). Initial molecular sputum diagnostics using Xpert MTB/RIF Ultra (Cepheid, https://www.cepheid.com) confirmed an infection with *Mycobacterium tuberculosis* with rifampin resistance. A line probe assay (Genotype MTBDR*plus* and MTBDR*sl*; Hain Lifescience, https://www.hain-lifescience.de) confirmed resistance to rifampin and additional resistance to isoniazid, whereas there was no resistance to fluoroquinolones. Sputum-smear microscopy demonstrated 3+ positive acid-fast bacilli (AFB). Rapid molecular drug-susceptibility testing (DST) was performed on DNA isolated from an initial positive *M. tuberculosis* culture using targeted next-generation sequencing Deeplex Myc-TB assay (Genoscreen, https://www.genoscreen.fr). We identified mutations katG S315T, rpoB L430P, and embB M306I, which indicated resistance to isoniazid, rifampin, and ethambutol (Figure 2, panel B). We confirmed the resistance pattern by phenotypic DST in mycobacterial growth indicator tube (MGIT; Becton Dickinson, https://www.bd.com) at the supranational reference TB laboratory (National Institute for Communicable Diseases, Johannesburg, South Africa).

In response to the patient’s extensive lung infiltration and high bacterial load, we initiated a regimen with 6 drugs: bedaquiline, linezolid, levofloxacin, cycloserine, clofazimine, and pyrazinamide (Figure 2, panel A). We ensured adherence by inpatient directly observed treatment. Clinical and microbiological response was slow; culture and smear microscopy results were negative once after 5 months of treatment (Figure 2, panel C). However, culture reversion occurred, and sustained culture conversion was never achieved despite clinical and transient radiologic improvement (Figure 1). Subsequent molecular DST based on targeted next-generation sequencing documented several frame shift mutations in *Rv0678* at position 779127 with 3.5% variant frequency, at 779130 with 16.0% frequency, and at 779407 with 27.8% frequency. Mutations in *rpoB*, *katG* and *embB* remained unchanged to baseline molecular DST. The de novo mutations were associated with phenotypic resistance to bedaquiline and clofazimine. All other drugs tested remained susceptible in molecular and phenotypic DST (Figure 2, panel B). We stopped bedaquiline and clofazimine administration and added 3 drugs, amikacin, meropenem/amoxicillin/clavulanic acid, and pretomanid, to maximize the probability of achieving conversion and cure. Treatment was ongoing as of February 2024.

## Conclusions

Bedaquiline has been shown to be a key drug for improving outcomes in MDR/RR TB patients (*4*). However, recent studies have demonstrated the emergence of bedaquiline resistance in patients failing MDR TB treatment, which, at the population level, points toward rapid bedaquiline resistance evolution and spread (*3*,*5*). Our results are particularly alarming because we demonstrated the evolution of bedaquiline resistance despite the use of an effective background regimen and well-documented adherence to treatment. This result is in line with the findings of recently published work from Mozambique, in which Barilar et al. demonstrated that bedaquiline resistance was found not only in *M. tuberculosis* strains resistant to fluoroquinolones but also in MDR or RR *M. tuberculosis* strains susceptible to other drugs used in the BPaL(M) regimen (*3*).

Taking all evidence together, the data suggest that current MDR/RR TB treatment regimens are unable to prevent the development of bedaquiline resistance in a subset of patients. A specific combination of pharmacokinetic and pharmacodynamic properties of the drug and pathogen or patient markers potentially result in rapid resistance development. Detecting 3 different *Rv0678* variants in the patient sample analyzed, an observation also made for several bedaquiline-resistant strains found previously (*3*,*6*), supports this observation.

In general, bedaquiline and clofazimine cross-resistance can result from underlying pretreatment resistance by infection with an already resistant strain, presence of heteroresistant strains and clonal populations, and de novo evolution of resistance during treatment (*5*). Here we demonstrate the rapid evolution and selection of several bedaquiline resistant subpopulations, despite resistance-appropriate treatment with 6 effective drugs. Our findings suggest a high bedaquiline resistance mutation rate that enables parallel emergence of different bedaquiline-resistant populations with different *Rv0678* mutations in a given patient. That finding is also supported by large-scale sequencing data obtained from patients with bedaquiline resistance in Mozambique (*3*).

In a recent meta-analysis, Mallik et al. reported acquired phenotypic bedaquiline resistance in 2.2%, genotypic resistance in 4.4% of cases (*5*), whereas Perumal et al. reported phenotypic resistance in 2.1% of cases (*7*). Future studies should further investigate mechanisms of bedaquiline resistance development, for example, to identify patients at risk. Bedaquiline seems to have a delayed bactericidal response, which could be a risk factor for drug resistance developing during early treatment, particularly in extensive disease (*8*). Some studies suggested the use of highly bactericidal companion drugs in combination with bedaquiline. Van Deun et al. reviewed the regimen composition on the basis of the concept of core drugs and companion drugs (*9*). In accordance with this strategy, our patient received 2 core drugs (bedaquiline and levofloxacin), 1 highly bactericidal companion drug (linezolid) and 2 highly sterilizing drugs (pyrazinamide and clofazimine); we added cycloserine. Despite strictly following van Deun’s concept of effective regimen composition, resistance to bedaquiline/clofazimine developed in the patient discussed here, who had severe lung destruction and high bacterial load. Derendinger et al. described acquired bedaquiline resistance in routine care in South Africa and considered use of <4 effective drugs, fluoroquinolone resistance, and previous or concurrent clofazimine use as risk factors for bedaquiline resistance (*6*). None of those factors were present in the case we describe.

Shao et al. showed in their population pharmocokinetic model that the current WHO recommended dosing of bedaquiline achieves a probability >90% of target attainment (*10*). However, Tanneau et al. proposed an exposure‒response relationship for bedaquiline, whereas the half-life of bacterial clearance was longer in pre–extensively drug-resistant (XDR) and XDR TB than in MDR TB. One might speculate that dose adjustments to bedaquiline favorably influence treatment outcomes (*11*), as has been done so far in individual cases applying therapeutic drug monitoring (*12*).

In conclusion, this case demonstrates the rapid evolution of phenotypic and genotypic resistance to bedaquiline and clofazimine, despite an effective individualized regimen. This finding is alarming because the BPaL(M) regimen may not be completely effective in an unknown proportion of patients. In particular, cases of extensive disease might be associated with a high risk for resistance and failure in real-world scenarios. We recommend further research into mechanisms of resistance, and prevention thereof, as well as rapid scale-up of DST capacity to identify and properly treat such cases as quickly as possible.
